# Adenosine deaminase augments SARS-CoV-2 specific cellular and humoral responses in aged mouse models of immunization and challenge

**DOI:** 10.3389/fimmu.2023.1138609

**Published:** 2023-03-14

**Authors:** Ebony N. Gary, Nicholas J. Tursi, Bryce M. Warner, Gina Cuismano, Jennifer Connors, Elizabeth M. Parzych, Bryan D. Griffin, Matthew R. Bell, Ali R. Ali, Drew Frase, Casey E. Hojecki, Gabriela A. Canziani, Irwin Chaiken, Toshitha Kannan, Estella Moffat, Carissa Embury-Hyatt, Sarah K. Wooton, Andrew Kossenkov, Ami Patel, Darwyn Kobasa, Michele A. Kutzler, Elias K. Haddad, David B. Weiner

**Affiliations:** ^1^ The Vaccine and Immunotherapy Center, The Wistar Institute, Philadelphia, PA, United States; ^2^ Perelman School of Medicine, University of Pennsylvania, Philadelphia, PA, United States; ^3^ Special Pathogens Program, National Microbiology Laboratory, Public Health Agency of Canada, Winnipeg, MB, Canada; ^4^ The Department of Medicine, Division of Infectious Diseases and HIV Medicine, Drexel University College of Medicine, Philadelphia, PA, United States; ^5^ The Department of Microbiology and Immunology, Drexel University College of Medicine, Philadelphia, PA, United States; ^6^ The Department of Biochemistry, Drexel University college of Medicine, Philadelphia, PA, United States; ^7^ The Genomics Core, The Wistar Institute, Philadelphia, PA, United States; ^8^ National Center for Foreign Animal Disease, Canadian Food Inspection Agency, Winnipeg, MB, Canada; ^9^ Ontario Veterinary College, University of Guelph, Guelph, ON, Canada; ^10^ Department of Medical Microbiology and Infectious Diseases, University of Manitoba, Winnipeg, MB, Canada

**Keywords:** age, SARS, DNA vaccine, adjuvant, adenosine deaminase (ADA)

## Abstract

Despite numerous clinically available vaccines and therapeutics, aged patients remain at increased risk for COVID-19 morbidity. Furthermore, various patient populations, including the aged can have suboptimal responses to SARS-CoV-2 vaccine antigens. Here, we characterized vaccine-induced responses to SARS-CoV-2 synthetic DNA vaccine antigens in aged mice. Aged mice exhibited altered cellular responses, including decreased IFNγ secretion and increased TNFα and IL-4 secretion suggestive of T_H_2-skewed responses. Aged mice exhibited decreased total binding and neutralizing antibodies in their serum but significantly increased T_H_2-type antigen-specific IgG1 antibody compared to their young counterparts. Strategies to enhance vaccine-induced immune responses are important, especially in aged patient populations. We observed that co-immunization with plasmid-encoded adenosine deaminase (pADA)enhanced immune responses in young animals. Ageing is associated with decreases in ADA function and expression. Here, we report that co-immunization with pADA enhanced IFNγ secretion while decreasing TNFα and IL-4 secretion. pADA expanded the breadth and affinity SARS-CoV-2 spike-specific antibodies while supporting T_H_1-type humoral responses in aged mice. scRNAseq analysis of aged lymph nodes revealed that pADA co-immunization supported a T_H_1 gene profile and decreased FoxP3 gene expression. Upon challenge, pADA co-immunization decreased viral loads in aged mice. These data support the use of mice as a model for age-associated decreased vaccine immunogenicity and infection-mediated morbidity and mortality in the context of SARS-CoV-2 vaccines and provide support for the use of adenosine deaminase as a molecular adjuvant in immune-challenged populations.

## Introduction

Severe acute respiratory syndrome coronavirus-2 (SARS-CoV-2), the virus which causes coronavirus disease of 2019 (COVID-19) continues to spread globally, having caused more than 600 million infections and over 6 million deaths to date. Aged patients have disproportionately increased COVID-19 morbidity and mortality (1). This increased disease severity is associated with increased immune infiltration in the lungs, a hallmark of the severe acute respiratory distress syndrome (ARDS) caused by SARS coronaviruses. Several vaccine candidates have been successfully deployed in the clinic, however, aged patients have significantly decreased vaccine-induced immunity, including decreased binding and neutralizing serum antibody titers, a decreased ability to neutralize emergent SARS-CoV-2 variants of concern (VOCs), and rapidly declining antibodies in serum (2–4). Thus, despite the overall high efficacy of available COVID-19 vaccines, aged patients remain at increased risk of severe COVID-19 and COVID-19-induced mortality.

Age-associated immune deficits are not unique to SARS-CoV-2. Aged patients have been reported to have decreased responses to several vaccines, including influenza (5–7)and hepatitis B virus (8, 9). However, the molecular mechanisms that underly decreased immune responses in aged individuals remains unclear. Mouse models of ageing display some of the hallmarks of age-associated immune deficiency including decreased serum antibody titers following immunization with vaccine or model antigens. This is theorized to be caused in part by aberrant germinal center function. Aged mice have been reported to have increased frequencies of follicular helper T cells (T_FH_) with decreased effector function coupled with increases in the suppressive capacity of follicular regulatory T cells (T_FR_) (10). Thus, improving T_FH_ cell function could restore age-associated impairment in humoral immunity and lead to more robust vaccine responses.

We determined that the enzyme adenosine deaminase-1 (ADA1) is a hallmark of the T_FH_ help program (11). Furthermore, ADA1 mutation causes severe-combined immunodeficiency in humans, characterized by broad T cell dysfunction. We have reported the ability of plasmid-encoded adenosine deaminase-1 (pADA) to enhance T_FH_ cell function and improve humoral responses to synthetic DNA (synDNA) antigens targeting HIV-1 (12) and SARS-CoV-2 (13). We recently reported that in young mice addition of pADA to a single DNA immunization supported increased antibody production and long-lived protection that was equal to two immunizations with unadjuvanted spike DNA vaccine (13). Additionally, ADA levels have been negatively correlated with age in human patients (14) and aged cultured human cells (15).

Here we evaluated ageing immune responses to a full-length SARS-CoV-2 spike DNA plasmid antigen (Wuhan D614G variant; pS) in young (6-8 week-old) and aged (68-72 week-old) mice. We observed significant impairment of cellular responses and T_H_2-skewed humoral responses to spike antigens in aged mice compared to their young counterparts. Aged mice were also significantly impaired in their ability to control SARS-CoV-2 replication in mouse challenge models. When aged animals were co-immunized twice with pADA we observed significant increases in vaccine-induced immunity resulting in cellular and humoral responses which were similar to those observed among twice-immunized young animals. Importantly, co-delivery of pADA enhanced both the magnitude and functional capacity of serum antibodies in aged mice. pADA co-immunization also supported enhanced antibody breadth and promoted long-term anti-SARS-CoV-2 functional antibody responses. Following challenge with mouse-adapted SARS-CoV-2 (MA-SARS2), aged mice immunized with pS alone had significantly higher viral loads as compared to pS-only immunized young mice. However, pADA co-immunization resulted in similarly lowered viral loads among young and aged animals.

To define the molecular mechanism of pADA-mediated immune enhancement and identify age-associated changes in immune function at the molecular level, we performed single-cell RNA sequencing (scRNAseq) on lymph nodes from young and aged mice immunized with pS alone or co-immunized with pS and pADA and evaluated changes in gene expression between aged and young mice. We observed increased inflammatory gene signatures with a concomitant increase in immunoregulatory genes among aged pS-only immunized mice compared to their matched young counterparts. When young and aged animals were co-immunized with pADA, these gene signatures were reversed with pADA co-immunized aged and young animals having similar expression of inflammatory and regulatory genes. We also observed an enhancement in T_H_2-like gene signatures among pS-only immunized aged animals while pADA co-immunization supported robust T_H_1-like gene signatures. These data define age-associated changes in lymphocyte gene expression and demonstrate that the genetic adjuvant pADA can enhance immune responses in aged mouse models of immunization and challenge.

## Results

### Cellular responses to spike antigens are decreased in aged mice

**Figure 1:**
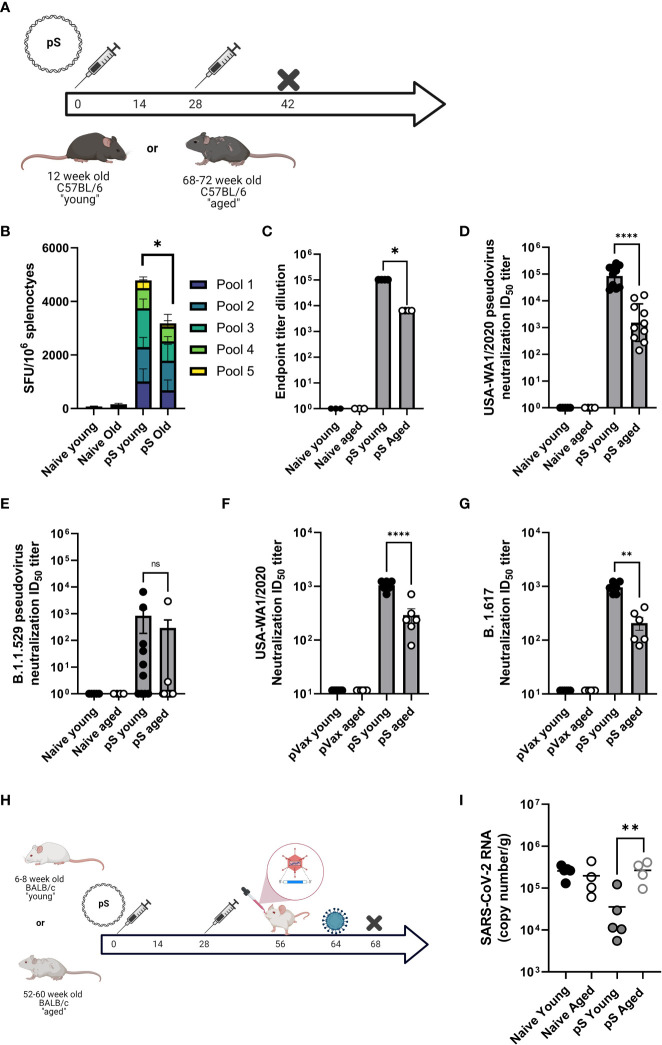
Aged mice display altered immune responses to SARS-CoV-2 DNA antigens. **(A)** 6-8 week-old “young” or 68-72 week-old “aged” C57BL/6 mice were immunized twice, separated by four weeks with 10ug of synthetic DNA plasmid encoding SARS-CoV-2 spike glycoprotein containing the D>G mutation at position 614 *via* electroporation and immune responses were assessed at day 14 post-2nd immunization. **(B)** IFNγ spot-forming units (SFU) in splenocytes as measured by ELISpot assay. SARS-CoV-2 parental spike RBD-specific endpoint titers in serum **(C)**. Serum pseudovirus neutralization against parental (USA-WA1/2020) **(D)** and Omicron VOC (B.1.1.529/BA.1) spike-pseudotyped viruses **(E)**. Serum live virus neutralization against parental (USA-WA1/2020) **(F)** and Delta VOC (B.1.617) spike-pseudotyped viruses **(G)**. **(H)** 6-8 week-old “young” or 52-60 week-old “aged” BALB/c mice were immunized as in A, rested, transduced intranasally with a human ACE2-expressing modified AAV vector, and challenged intranasally with 1x105 PFU of parental SARS-CoV-2 (VIDO-01). Viral load was quantified in the lungs at day 4 post-challenge by qPCR **(I)**. Bars represent the mean, symbols represent the mean of duplicate assay per animal, and error bars represent the SEM. Data are representative of two **(A–G)** or one independent experiment **(H, I)** with N= 5-10 mice per group. ns, not significant. *P<0.05, **P<0.01, and ****P<0.0001 by Kruskal-Wallis ANOVA.

In the context of immunization, ageing is associated with decreased cellular responses in both preclinical models and in patients. Therefore, we compared cellular responses to full-length spike glycoprotein DNA immunogens (**pS**) in aged (68–72-week-old) and young (6-12 week old) mice. Mice were immunized twice, separated by four weeks with 10ug of DNA plasmid encoding the full-length spike glycoprotein of SARS-CoV-2 (**pS**) containing the D>G mutation at position 614 (**Figure 1A**), or immunized with 10ug of empty plasmid vector (pVax) and cellular responses were evaluated by IFNγ ELISpot following stimulation with matched spike peptides. Aged animals had significantly fewer IFNγ spot-forming units (SFUs) in their spleens (**Figure 1B**) compared to their young counterparts suggesting an age-associated decrease in cellular immune responses to SARS-CoV-2 antigens.

### Aged mice display decreased humoral responses to spike DNA antigens

It has been previously reported that aged patients have decreased antibody responses to vaccine antigens. mRNA vaccinated aged patients develop decreased SARS-CoV-2 specific antibody responses compared to young patients. These decreased levels of binding antibodies translate to decreased SARS-CoV-2 neutralizing antibodies, a faster decline in neutralization titer post-vaccination, and a decreased ability to neutralize SARS-CoV-2 variants of concern in aged patients as compared to young-adult patients (2, 3). Therefore, we determined if this age-associated humoral deficit could be modeled in mice. Mice were immunized twice, as described above and antibody responses were defined *via* binding ELISA, pseudovirus, and live virus neutralization. After two immunizations we detected robust spike receptor-binding domain (RBD) specific IgG in the serum of young mice, however aged animals had significantly less RBD-specific antibody in their sera (**Figure 1C**). Sera from aged animals was impaired in the ability to neutralize pseudotyped viruses representing the Washington variant (USA-WA1/2020) or the Omicron variant (B.1.1.529) (**Figures 1D, E**). We also observed a significant age-associate defect in the ability of immune mouse sera to neutralize live USA-WA1/202 and Delta variant (B.1.617) viruses (**Figures 1F, G**). These data demonstrate that aged mice are a reliable model for studying age-associated humoral deficits to SARS-CoV-2 vaccine antigens and model the clinical observation of decreased quantity and quality of humoral immunity among elderly SARS-CoV-2 vaccine recipients.

### Aged mice have increased viral burden following challenge

We reported on the utility of a modified adeno-associated virus-6 (AAV6.2FF) expressing human angiotensin-converting enzyme-2 (AAV6.2FF hACE2) to transduce the lungs of wild type mice as a model for SARS-CoV-2 infection in wild-type mice (16). We immunized young (6-8 week-old) and aged (52-60 week-old) BALB/c mice twice with 10ug of pS. Following rest, animals were transduced with hACE2 *via* intranasal instillation of AAV6.2FF hACE2 and infected with 10e5 PFU of SARS-CoV-2 seven days post transduction (**Figure 1H**). Four days after infection, viral loads were quantified in the lungs *via* qPCR. Naïve animals had similarly high viral loads, independent of age. Immunization reduced viral loads in young mice, however aged immunized animals had significantly higher viral loads than their immunized young counterparts, with levels equivalent to unimmunized animals (**Figure 1I**). These data suggest that the age-associated immune impairment observed following immunization can have a significant impact on challenge outcome in the mouse model. Further, it suggests that aged mice may recapitulate the immune deficits observed in patients and provides a model system for studying SARS-CoV-2 vaccines, therapeutics, and infection in the context of an ageing immune system.

### pADA co-immunization rescues age-associated deficits in T cell responses to SARS-CoV-DNA antigens

**Figure 2:**
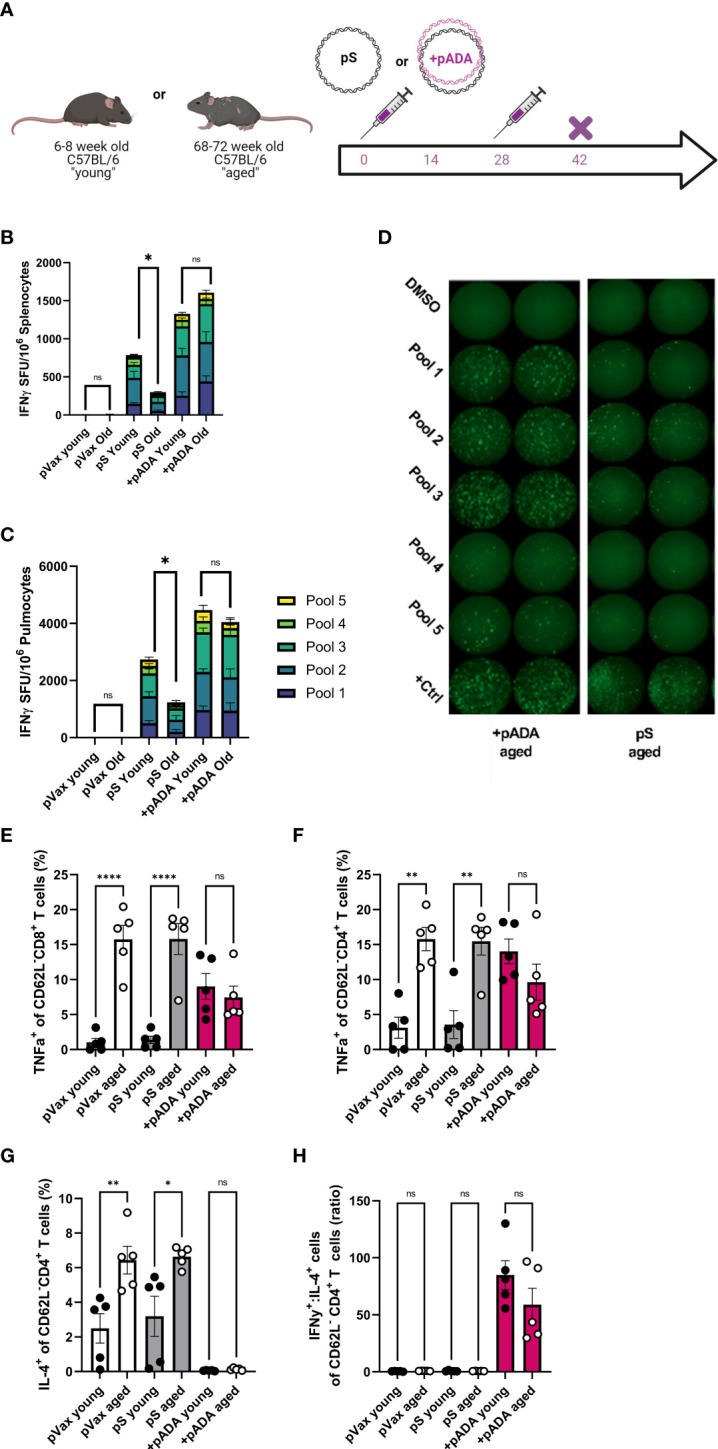
pADA co-immunization supports enhanced antigen-specific effector function and inhibits age-associated inflammatory cytokine secretion. Mice were immunized with 10ug of spike-encoding plasmid DNA (pS) or co-immunized with 10ug od pS and 10ug of DNA plasmid encoding mouse adenosine deaminase (+pADA) twice, separated by four weeks and cellular responses were evaluated at day 14 post-second immunization **(A)**. Interferon gamma spot-forming units in spleens **(B)** and lungs **(C)**; representative IFNγ wells **(D)** as measured by ELISpot assay. Frequency of TNFα^+^ CD8^+^ **(E)** and CD4^+^ T cells in spleens **(F)**. Frequency of IL-4+ CD4^+^ T cells **(G)**, and the ratio of IFNγ^+^ to IL-4^+^ CD8^+^ T cells **(H)**. Data are representative of three independent experiments with N=5 per group **(B, C)** or one experiment with N=5/group **(E–H)**. Symbols represent individual animals, bars represent the mean, error bars represent the standard deviations. ns, not significant. *P<0.05, **P<0.01, and ****P<0.0001 by Mann-Whitney-U test.

Adenosine deaminase-1 (ADA1) deaminates adenosine to produce inosine and is essential for lymphocyte homeostasis (17). Recently we reported that co-immunization with plasmid-encoded adenosine deaminase (pADA) enhanced cellular responses to pS in young mice (13). Therefore, we quantified SARS-CoV-2 spike-specific cell-mediated immunity by both IFNγ fluorospot assay and intracellular cytokine staining (ICS) following pADA co-administration in aged mice. Mice were immunized twice separated by four weeks with pS alone, co-immunized with pS and pADA, or immunized with 20ug of empty plasmid vector (pVAX) (**Figure 2A**). Aged animals had significant decreases in IFNγ spot forming units (SFUs) in their spleens (**Figure 2B**) and lungs (**Figures 2C, D**) compared to their young counterparts when immunized with pS alone. However, pADA co-immunization significantly enhanced spike specific IFNy secretion in young and aged spleens and lungs (**Figures 2B, C**). Notably, in contrast to the significant differences observed between young and aged animals immunized with pS alone, there was no significant difference in IFNγ secretion in the lungs or spleens between young and aged pADA co-immunized animals. This data suggests that pADA co-immunization supports enhancement of antigen-specific T cell responses and can rescue age-associated defects in IFNγ secretion post-immunization.

We also evaluated T cell effector function in these animals by ICS. Following stimulation with spike peptide pools, we evaluated the frequencies of IFNγ^+^ and TNFα^+^ CD8^+^ and IFNγ^+^, TNFα^+^, and IL-2^+^ CD4^+^ T cells in the spleens of immunized mice in both age groups (**Supplementary Figure 1**). TNFα^+^ CD8^+^ T cells were significantly increased in aged animals independent of immunization group. Interestingly, pADA co-immunization significantly increased the frequencies of TNFα^+^ CD8^+^ T cells in the spleens of young mice compared to pS-only immunized young mice while decreasing the frequency of TNFα^+^ CD8^+^ T cells in the spleens of pADA co-immunized aged mice compared to pS-only immunized aged mice, such that there was no significant difference in the frequency of TNFα^+^ CD8^+^ splenocytes between pADA co-immunized young and aged mice (**Figure 2E**). TNFα is a well-characterized proinflammatory molecule associated with age-associated pathogenic inflammation (inflammaging) (18). This pattern was also evident among CD4^+^ T cells (**Figure 2F**).

The age-associated increases in TNFα suggested that aged T cells are predisposed to a proinflammatory phenotype. Therefore, we also measured the frequency of IL-4^+^ CD4^+^ (**Figure 2G**) T cells and compared the ratio of IFNy^+^ cells to IL-4^+^ cells (**Figure 2H**) to evaluate the T_H_1 or T_H_2 biasing. Aged animals had significantly more IL-4^+^ CD4^+^ T cells than young animals however, pADA co-administration drastically decreased IL-4 secretion in both young and aged animals (**Figure 2G**) and significantly increased the ratio of IFNγ:IL-4^+^ CD4^+^ T cells (**Figure 2H**), suggesting a strong T_H_1 bias among CD4^+^ T cells in the presence of pADA. Together, these data suggest that pADA co-immunization can ameliorate age associated proinflammatory cytokine secretion from CD4^+^ and CD8^+^ T cells while supporting antigen-specific effector cytokine secretion.

### pADA enhances acute SARS-CoV-spike-specific humoral responses in aged mice and supports T_H_1-biased antibody production

*ADA1* expression identifies GC T_FH_ cells (11). We previously reported that co-delivery of pADA enhanced anti-SARS-CoV-2 humoral responses in young mice and supported single-dose protection from challenge at acute and memory timepoints (13). We confirmed that a single pADA co-immunization robustly increases SARS-CoV-2 serum antibodies (**Supplementary Figures 2A, B**) however, following two immunizations, pS-only and pADA co-immunized young mice had similar quantities of binding antibodies in their sera (**Supplementary Figures 2A, C**). We compared humoral responses in young and aged mice after one or two doses of pS alone or pS and pADA and evaluated antibody responses after each immunization. Following a single immunization with pS alone, only one of eight aged animals seroconverted, while ~50% of pADA co-immunized animals seroconverted. (**Supplementary Figures 2D, E**). Even after two immunizations many aged animals immunized with pS alone had not seroconverted and we observed a significant increase in the ability of serum from pADA co-immunized aged mice to neutralize pseudotyped viruses compared to pS-only immunized aged mice (**Supplementary Figure 2F**). Thus we performed all ageing experiments using a prime-boost regimen.

**Figure 3:**
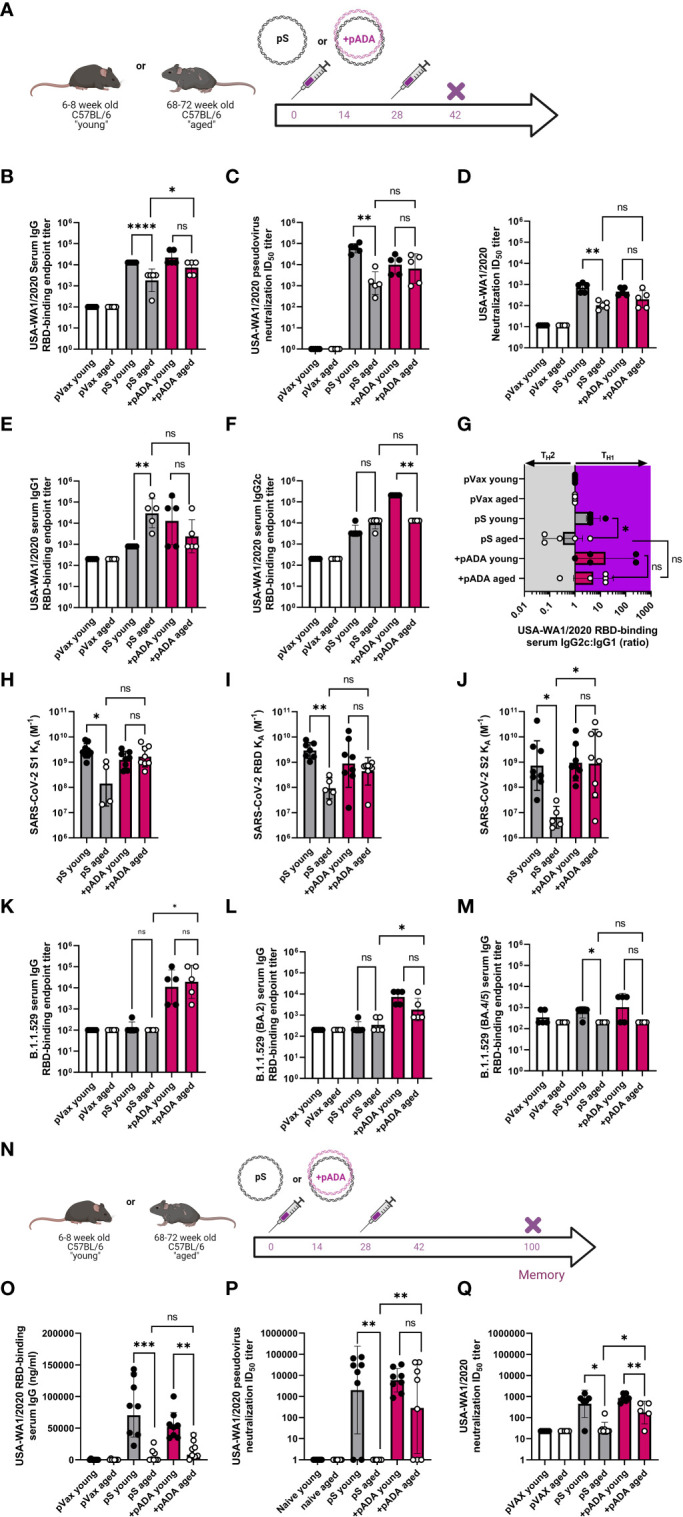
pADA co-immunization enhances SARS-CoV-2 humoral responses and promotes long-lived antibody responses in aged mice. Mice were immunized as in **Figure 2** and antibody responses were evaluated 12-14 days post-2^nd^ immunization **(A)**. **(B)** Serum binding, **(C)** pseudovirus neutralization, and **(D)** live virus neutralization against SARS-CoV-2 parental (USA-WA1/2020). Serum binding IgG1 **(E)**, IgG2c **(F)**, and the ratio of IgG2c:IgG1 **(G)**. Serum antibody affinity as measured by surface plasmon resonance against parental SARS-CoV-2 S1 **(H)**, RBD **(I)**, and S2 **(J)** spike subunits. Omicron (B.1.529/BA.1) **(K)**, BA.2 **(L)** and BA.4/5 **(M)** RBD Serum binding endpoint titers. Mice were immunized twice separated by four weeks with pS alone or co-immunized with pS and pADA as in A, and antibody responses were evaluated 72 days post-2nd immunization **(N)**. **(O)** Serum binding, **(P)** pseudovirus neutralization, and **(Q)** live virus neutralization against SARS-CoV-2 parental (USA-WA1/2020) at 6072 days post second immunization. Data are representative of one experiment. Symbols represent the mean of duplicate assays for individual animals, bars represent the geometric mean, error bars represent the geometric standard deviation. *P<0.05, **P<0.01, ***P<0.001, and ****P<0.0001 by Mann-Whitney-U test.

Mice were immunized twice with pS alone or co-immunized with 10ug of pS and 10ug of pADA and sacrificed at day 14 post-2nd immunization or day 60 post-2^nd^ immunization (**Figure 3A**). As previously observed, when immunized with pS alone, aged mice had statistically significant decreases in spike-binding, pseudovirus neutralizing, and live virus neutralizing antibody in their serum compared to pS immunized young mice (**Figures 3B–D**). Among young animals, two immunizations with pADA was not significantly different from two immunizations with pS alone. We observed increases in spike-specific antibody amongst aged animals such that we could not detect statistical differences in antibody quantity young and aged animals co-immunized pADA (**Figures 3B, D**). We observed a significant increase in binding antibody between pADA co-immunized aged mice and their pS-only immunized aged counterparts (**Figure 3B**), but no significant differences were observed when we compared the pseudovirus or live-virus neutralization capacity between pS-only and pADA co-immunized aged animals (**Figures 3C, D**). When we evaluated T_H_2 versus T_H_1-type antibody responses by measuring RBD-binding IgG1 (**Figure 3E**) and IgG2c (**Figure 3F**) respectively, we observed increased RBD-specific IgG1 in the serum of aged mice when immunized with pS alone (**Figure 3E**). This resulted in a decreased IgG2c:IgG1 ratio among aged pS-only immunized mice (**Figure 3G**). pADA co-immunization supported increased IgG2c production and increased the IgG2c:IgG1 ratio in both young and aged mice (**Figure 3G**). These data suggest that pADA co-immunization supports a T_H_1-type humoral response in aged mice.

We measured the affinity of sera antibodies for SARS-CoV-2 parental spike using SPR detection. We observed a low binding affinity (expressed as the equilibrium association constant, KA M^-1^) in the serum antibodies of pS-only immunized aged mice compared to their young counterparts across the entirety of the spike protein (**Figures 3H–J**). pADA co-immunization resulted in a trend toward enhanced antibody affinity against all spike domain proteins such that young and aged pADA co-immunized mouse sera had equal binding affinity (**Figures 3H–J**). Binding to the S2 domain was significantly enhanced in pADA co-immunized aged mice as compared to pS-only immunized mice (**Figure 3J**). To determine if pADA co-immunization enhances antibody breadth against SARS-CoV-2 variants of concern (VOCs), we quantified the ability of these sera to bind or neutralize Omicron subvariants. Strikingly, only sera from pADA co-immunized mice were capable of binding BA.1 RBD and sera from young and aged animals bound BA.1 RBD to similar levels (**Figure 3K**). Similarly, we observed minimal binding to BA.2 RBD from young or aged pS-only immunized sera. However, pADA co-immunization enhanced BA.2 binding capacity in young and aged sera (**Figure 3L**). When we quantified binding to BA.4/BA.5 RBD, pADA co-immunization modestly enhanced the capacity of young sera to bind BA.4/5 RBD (**Figure 3M**). These data indicate that pADA co-immunization enhances humoral responses against matched SARS-CoV-2 variants and may support increased antibody breadth against VOCs.

When we evaluated humoral responses at a memory (>60 days post-2^nd^ immunization) timepoint (**Figure 3N**), all immunized aged mice had decreases in RBD-binding serum IgG compared to young mice and we observed no significant differences in the quantity of binding antibodies in the serum of pS-only immunized aged animals and pADA co-immunized aged animals (**Figure 3O**). Sera from pS-only immunized aged mice had no detectable pseudovirus (**Figure 3P**), and minimal live virus (**Figure 3Q**) neutralizing capacity. However, sera from pADA co-immunized aged animals displayed significant increases in the capacity to neutralize pseudovirus (**Figure 3P**) and live virus (**Figure 3Q**) at this memory timepoint compared to pS-only immunized aged animals. These data demonstrate that pADA co-immunization can enhance the magnitude, duration, and function of antigen-specific humoral responses in aged mice and suggest pADA-mediated enhancement of antibody function.

Germinal center T_FH_ cells instruct the development of high affinity antibody and antigen-specific memory B cells. We measured the frequency of T_FH_ cells in the lymph nodes of young and aged mice after two immunizations with pS alone or co-immunization with pADA. In keeping with previous reports of aged lymph node GC populations, we observed a significant increase in GC T_FH_ in the lymph nodes of aged animals as compared to young animals, independent of immunization group (**Supplementary Figures 3A, B**). While there was a trend toward increased T_FH_ in the lymph nodes of pADA co-immunized young mice compared to pS immunized young animals, it was not significant. However, among aged animals, pADA co-immunization significantly increased the frequency of lymph node GC T_FH_ compared to empty plasmid, or pS only immunized aged animals (**Supplementary Figures 4B**). Conversely, ageing was associated with decreases in the frequency of AIM^+^ T_FH_ in the lymph nodes of animals despite pADA co-immunization (**Supplementary Figure 4C**). These data suggest that age-associated changes in germinal center cell frequencies may play a role in the decreased humoral responses observed among aged mice and that pADA may support increased GC function *in vivo*.

### pADA co-immunization enhances protection in mouse models of SARS-CoV-infection

**Figure 4:**
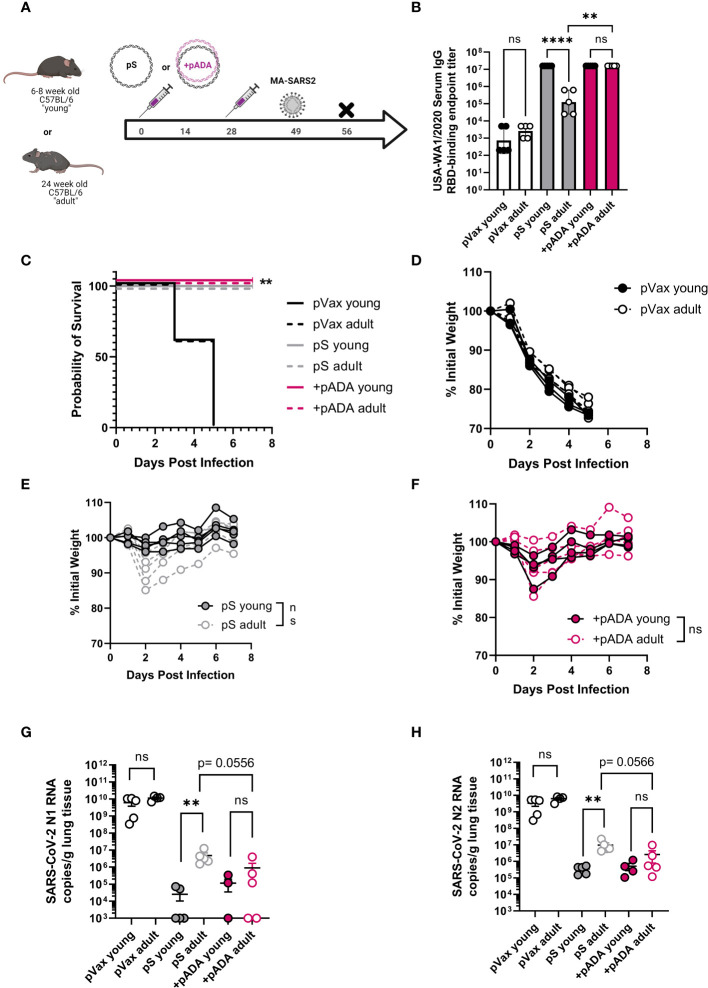
pADA enhances protection from mouse-adapted SARS-CoV-2 challenge in aged mice. Young (6-12 week-old, black circles), and adult (24-30 week-old, gray-filled circles) C57BL/6 mice were immunized twice as in **Figure 2** and challenged with 1x10^5^ plaque-forming units of mouse-adapted SARS-CoV-2 (MA-SAR2) on day 19 post-challenge and morbidity and mortality were monitored until day 7 post-challenge **(A)**. Parental SARS-CoV-2 (USA-WA1/2020) receptor-binding domain (RBD)-specific IgG endpoint titers at day 10 post-2^nd^ immunization **(B)**. Survival curves for all animals **(C)**, and weight loss among empty plasmid immunized (pVax) **(D)**, pS-only immunized **(E)**, and pADA co-immunized **(F)** mice. SARS-CoV-2 nucleoprotein N1 **(G)** and N2 **(H)** RNA in the lungs of animals at day 7 post-challenge. Data are representative of a single experiment with N=5 (12 and 24 wk-old) and N=10 (66-68wk old) mice per group. Symbols represent the mean of duplicate **(B)** or triplicate **(G, H)** assays for individual animals, bars represent group mean and error bars represent the SEM. Symbols represent individual animals **(C–F)**. ns, not significant. **P<0.01 and ****P<0.0001 by Kruskall-Wallis ANOVA **(C)** or Mann-Whitney-U test between indicated groups **(B, D–H)**.

Previous reports of challenge with MA-SARS2 isolates have indicated that mice as young as 24 weeks of age may be at increased susceptibility to SARS-CoV-2 induced morbidity and mortality (19). We therefore immunized young (6-8 week-old) and “adult” (24 week-old) mice twice, separated by four weeks with pS alone (young and adult), or co-immunized them with pS and pADA (+pADA). Approximately 20 days post final immunization, animals were challenged intranasally with 1x10^5^ PFU of MA-SARS2 (**Figure 4A**). We evaluated RBD-binding IgG in serum at day 14 post-2^nd^ immunization and confirmed that pS immunized adult mice had decreased RBD-binding IgG in their sera and that pADA co-immunization supported equivalent RBD binding IgG in the sera of young and adult mice (**Figure 4B**). MA-SARS2 challenge is lethal, and all unimmunized animals succumbed to infection by day 5 post-challenge (**Figures 4C, D**). All immunized young and adult animals survived infection (**Figure 4C**). Adult pS immunized mice had a trend toward increased weight loss compared to young pS immunized mice but this was not statistically significant (**Figure 4E**). Among pADA co-immunized animals young and adult mice had similar morbidity (**Figure 4F**). We collected the lungs from all animals at euthanasia and evaluated SARS-CoV-2 viral load *via* qPCR for nucleocapsid (N1 and N2) RNA. All immunized animals, independent of age displayed lowered viral loads (**Figures 4G, H**). In support of our observations, pS only immunized adult animals had higher levels of viral RNA in their lungs compared to their young counterparts (**Figures 4G, H**). Among pADA co-immunized mice, we detected no significant differences in viral loads between young and aged mice (**Figures 4G, H**). We observed a trend toward decreased viral loads between pS-only and pADA co-immunized aged mice (**Figures 4G, H**).

**Figure 5:**
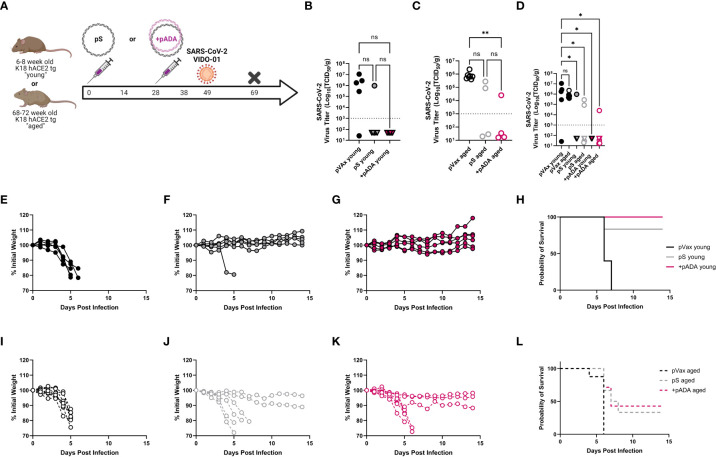
pADA co-immunization supports protection in a human ACE2tg aging mouse SARS-CoV-2 challenge model. Young (6-12 week-old) or aged (52-68 week-old) human ACE2 transgenic mice were immunized twice as in **Figure 2** and challenged with 1x10^5^ plaque-forming units of SARS-CoV-2 (VIDO-01) on day 19 post-challenge and morbidity and mortality were monitored until day 15 post-challenge **(A)**. SARS-CoV-2 lung viral loads as measured by TCID50 assay among young **(B)**, aged **(C)**, and **(D)** all mice including survivors(triangles) and non-survivors (circles). Weight loss among empty-plasmid (pVax) **(E)**, pS-only **(F)**, and pADA co-immunized **(G)** young animals, and overall survival among young animals **(H)**. Weight loss among empty-plasmid (pVax) **(I)**, pS-only **(J)**, and pADA co-immunized **(K)** aged animals, and overall survival among aged animals **(L)**. Symbols represent the mean of triplicate assays for individual animals **(B–D)** or weights of individual animals **(E–G, I–K)**. Lines represent the group probability of survival **(H, L)**. ns, not significant. *P<0.05 and **P<0.01, by Kruskal-Walis ANOVA.

We also challenged young and aged human ACE2 transgenic (**K18 hACE2tg**) mice. These mice are the current standard for mouse SARS-CoV-2 challenge in young mice, however there is an understandably limited supply of these animals at advanced age. Young (6-8 week-old) and aged (68-72 week-old) K18 hACE2tg mice were immunized twice separated by four weeks with pS alone or co-immunized with pS and pADA, and challenged 21 days post 2^nd^ immunization with 1x10^5^ PFU of SARS-CoV-2 (VIDO-01, parental). Survival was monitored for 15 days post-challenge (**Figure 5A**). We collected lungs to quantify viral loads at the time of euthanasia. As expected, all surviving animals had undetectable viral loads (**Figures 5B, D**, triangles). When we evaluated viral loads in young animals, we observed no difference between pS and pADA co-immunized animals as expected, while both immunization groups lowered average viral load **(** **Figure 5B**). Among aged mice, only animals co-immunized with pADA had statistically significantly lowered viral loads compared to unimmunized aged mice (**Figure 5C**). While viral loads among pS and pADA co-immunized animals were not statistically significant, only one of four pADA co-immunized animals had detectable viral loads in their lungs at the time of euthanasia compared to two of four pS-only immunized aged animals (**Figure 5C**). When survivors and non-survivor (circles) viral loads were evaluated together, all immunized mice had statistically significantly lowered viral loads (**Figure 5D**). As we observed in the MA-SARS2 challenge model, unimmunized animals lost significant weight independent of age group (**Figures 5E, I**) and succumbed to infection within the first week (**Figures 5H, L**). Among young animals, a single pS-only immunized mouse lost weight and succumbed to infection (**Figures 5F, H**). Young pADA co-immunized mice were 100% protected from morbidity and mortality (**Figures 5G, H**). Aged animals displayed increased morbidity and mortality in both immunization groups (**Figures 5J, K**). Among pS-only immunized aged mice, 4 of 6 animals succumbed to infection (**Figures 5J, L**), while pADA co-immunized aged animals lost weight and of 6 succumbed to infection (**Figures 5K, L**). Together, these data suggest that pADA co-immunization can significantly impact post-challenge viral loads in aged mice.

### Lymph node gene expression identifies a molecular phenotype of aged immune responses which can be altered by pADA co-delivery

To further define the molecular mechanisms underpinning the changes in immunogenicity in young and aged mice as well as identify the molecular mechanisms of pADA-mediated enhancement, we performed single-cell RNA sequencing (scRNAseq) of lymph node immune cells after two immunizations with pS alone or pS and pADA in young and aged C57BL/6 animals. Seurat clustering identified 12 distinct clusters (**Supplementary Figures 4A, B**). The majority of sequenced cells were B or T lymphocytes as determined by SingleR package analysis (**Supplementary Figure 4B**). We then further resolved T cells into sub-types based on gene markers from each cluster. Cluster 2 was considered as CD8^+^ T cells, while clusters 3, 4, and 5, predominantly contained CD4^+^ T cells. Re-clustering the CD4+ T cells further helped identify Foxp3+ regulatory T cells and a subset of follicular T helper cells (Foxp3^-^ CXCR5^+^ PD1^+^ Bcl6^+^) among other T cell sub-types (**Supplementary Figure 4C**)

**Figure 6:**
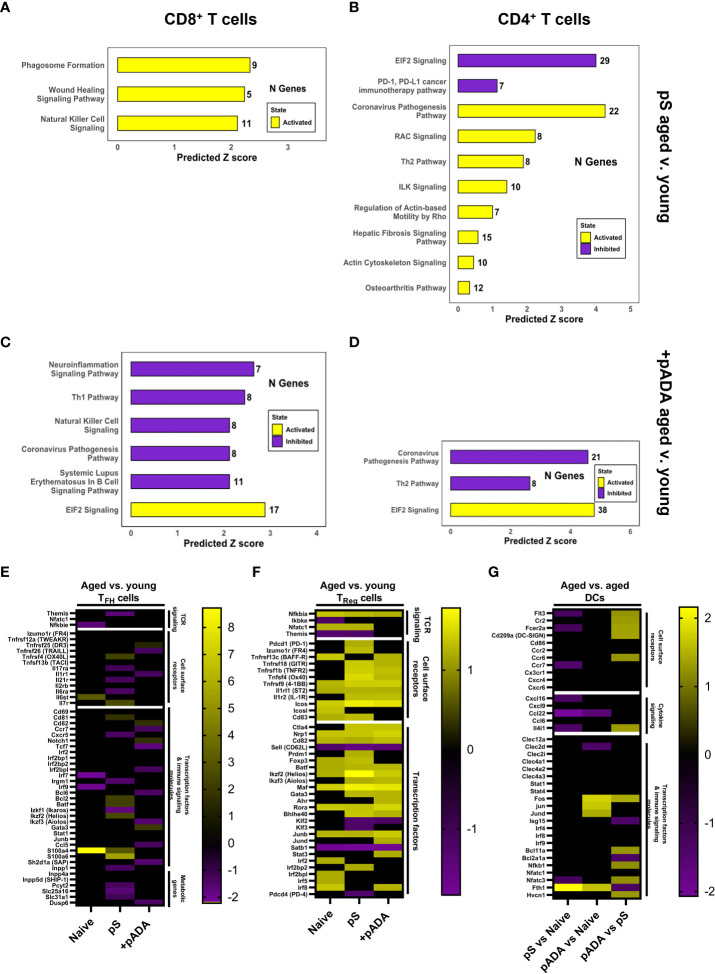
pADA co-immunization corrects age-associated increases in proinflammatory and pro-regulatory gene signatures. Mice were immunized twice with pS alone or co-immunized with pADA as in **Figure 2** and popliteal and inguinal lymph nodes from 10 mice per immunization condition were pooled and subjected to single-cell RNA sequencing (scRNAseq) and ingenuity pathway analysis (IPA). IPA analysis of differentially regulated pathways in aged lymph nodes as compared to young lymph nodes following pS-only immunization among CD8^+^ T cells **(A)** and CD4^+^ T cells **(B)**. IPA analysis of differentially regulated pathways in aged lymph nodes as compared to young lymph nodes following pS and pADA co-immunization among CD8^+^ T cells **(C)** and CD4^+^ T cells **(D)**. **(E)** Select differentially expressed genes in aged animals compared to young animals among follicular helper T (T_FH_) cells. **(F)** Select differentially expressed genes in aged animals compared to young animals among T regulatory (T_Reg_) cells. **(G)** Select differentially expressed genes in the dendritic cell (DC) compartment of aged animals among immunization groups.

We performed ingenuity pathway analysis (**IPA**) on the CD4^+^ and CD8^+^ T cell compartments and compared global changes in immune pathways between young and aged animals following pS-only or pADA co-immunization. Among CD8^+^ T cells, lymph nodes from aged animals immunized with pS alone activated (yellow bars) pathways associated with phagosome formation, wound healing, and natural killer cell signaling. These pathways include many inflammatory genes such as IFNγ receptors and IL-18 receptor components (**Figure 6A**). Conversely CD8^+^ T cells from aged mice co-immunized with pADA displayed inhibited (purple bars) pro-inflammatory pathways including natural killer cell signaling, neuroinflammation signaling, SLE B cell signaling, T_H_1 signaling, and the coronavirus pathogenesis pathway which included *CCL5* and *CCR2* and numerous ribosomal subunit proteins (**Figure 6C**). pADA co-immunized CD8^+^ T cells significantly activated a single pathway for EIF2 signaling. Eukaryotic initiation factor 2 (EIF2) has been implicated in protective proinflammatory responses and is required for control of intracellular bacterial infection (20). In the CD4^+^ T cell compartment we observed similar patterns of increased inflammatory pathway activation among aged pS-only immunized animals compared to pS-only immunized young animals. Activated pathways in this compartment included the coronavirus pathogenesis pathway and T_H_2 signaling pathways (**Figure 6B**). EIF2 signaling was significantly decreased in aged pS-only immunized lymph nodes as was the PD-1/PD-L1 signaling pathway which is essential for T_FH_ cell differentiation and the generation of regulatory follicular helper cells (**T_FR_**) (21) (**Figure 6B**). Conversely when aged animals were co-immunized with pADA, the EIF2 signaling pathway was activated while the coronavirus pathogenesis and T_H_2 signaling pathways were deactivated (**Figure 6D**). Together these data suggest that the aged lymph node is characterized by increased inflammatory gene expression which can be augmented by ADA and confirm our observation of strong T_H_2 skewing when aged animals are immunized with pS alone.

pADA co-immunization resulted in similar levels of binding and neutralizing serum antibodies in young and aged animals (**Figure 3**) and increased the frequencies of lymph node GC T_FH_ in aged animals (**Supplementary Figure 3**). Therefore, we interrogated T_FH_ gene expression *via* scRNAseq. scRNAseq identified T_FH_ cells as a subpopulation of the CD4^+^ T cell cluster. We confirmed the expression of established T_FH_ markers within this compartment including *Izumo1r* (folate receptor-4, FR4) (22) and *Icos* (23) (**Figure 6E**). We compared DEGs among T_FH_ cells in aged versus young animals under naïve, pS-only, and pADA co-immunized conditions. When animals were immunized with pS only, we detected decreased expression of interferon-inducible immunity related genes (*Irgm1*) which supports the expansion and survival of mature effector CD4^+^ T cells (24) and promotes a T_H_1-type IFNγ producing T cell population and inhibits T_H_17 inflammatory-type cytokine secretion (25). We also observed decreased expression of Ikaros zinc finger family group protein 1 (*Izkf1*) which has been shown to support STAT3 -mediated Bcl6-mediated T_FH_ differentiation (26). We observed decreased expression of the IL-21 receptor*(Il21r*) which critically supports T_FH_ differentiation and function (27). THEMIS (*Themis*) expression was also decreased in T_FH_ of aged pS-immunized mice compared to young animals. Decreased THEMIS expression is associated with decreased regulatory capacity of T cells (28). In pS-only immunized mice, compared to their young counterparts we observed decreased expression of CXCR5, which is the canonical T_FH_ chemokine receptor and essential for GC entry. Decreased CXCR5 expression was associated with decreased expression of phosphate cytidylyltransferase 2 (*Pcyt2*), a critical component of phosphatidylethanolamine (PE) synthesis which supports sustained surface expression of CXCR5 on T_FH_ cells (29) (**Figure 6E**). Together these suggest a T_H_17-type GC with increased regulatory function in the lymph nodes of aged mice which supports our observed decreased humoral responses in pS-only immunized aged animals.

We investigated DEGs in the regulatory T cell (T_Reg_) compartment in the lymph node. Aged animals had increased expression of CD83, which is critical for Treg differentiation and stability (30) (**Figure 6F**). The Treg master transcription factor, FOXP3 (*foxp3*) (31) was increased among the Tregs of pS and naïve aged mice compared to their young counterparts but was unchanged between pADA co-immunized aged animals and pADA co-immunized young animals. This gene expression profile suggests and pADA-associated decrease in T_Reg_ cell function and supports our observations of decreased frequencies of T_Reg_ in pADA co-immunized lymph nodes.

We previously reported that treatment of human monocyte-derived dendritic cells (mDCs) with recombinant ADA-1 *in vitro* supported a balance pro- and anti-inflammatory cytokine secretion profile from these cells which included increased production of IL-6 which is critical to T_FH_ differentiation (32). The observed decreases in both cellular and humoral responses observed in aged mice in these experiments suggested that age-associated dendritic cell (DC) dysfunction may play a role. We compared DEGs in LN DCs between pS-only and pADA co-immunized aged mice. The Fc epsilon receptor 2A (Fcer2a) was increased in the dendritic cell compartment of pADA co-immunized aged animals compared to pS only immunized aged animals (**Figure 6G**). These data suggest DC presentation of immune complexes to B cells in aged lymph nodes is impaired and that pADA co-delivery with antigen can enhance this DC phenotype *in vivo*. In support of enhanced DC function in the presence of pADA, we observed increased expression of FMS-like tyrosine kinase 3 (FLT3, *Flt3*). FLT3 expression is indispensable for plasmacytoid (pDC) and conventional dendritic cell (cDC) development (33) and is associated with type I IFN secretion by DCs (34). We also observed increased expression of CD209a, the mouse ortholog of DC-SIGN, which supports robust CD8^+^ T cell adaptive immunity *in vivo (* 35). pADA co-immunized aged LN DCs had increased expression of the transcription factor Bcl11a, which is critical for pDC development (36), coupled with significant decreases in the Bcl2a1a transcription factor, which supports cDC development (37). This data suggests that pADA co-immunization supports pDC differentiation and function.

## Discussion

The currently licensed SARS-CoV-2 vaccines induce robust immunity in younger populations however, aged individuals exhibit decreased antibody responses compared to their younger counterparts. Elderly patients are at increased risk of COVID-19 morbidity and mortality. Thus, identifying the mechanisms underpinning age-associated deficiencies in immune responses to vaccine antigens and the development of vaccine platforms and modalities which enhance immune responses in aged patients is critical. We observed significantly impaired humoral responses in aged mice compared to their young counterparts, recapitulating what has been reported in humans (2, 38–40). Aged mice also displayed differences in cellular responses, including decreased IFNγ secretion, which has been reported in human patients (41).

Here we determined that pADA co-delivery enhanced cellular and humoral responses and supported a strong T_H_1-biased immunity in ageing mouse models of SARS-CoV-2 synthetic DNA vaccination. We characterized for the first time the ageing mouse immune response to synDNA antigens *in vivo* and confirmed that aged mice recapitulate the decreased cellular and humoral responses observed in patients. Importantly, when aged mice were co-immunized with pADA, they displayed cellular and humoral responses on par with their young counterparts. In the CD8^+^ T cell compartment we observed that aged animals showed increased secretion of TNFα independent of immunization. TNFα is a characteristic cytokine associated with inflammaging (42–44). Interestingly, pADA co-immunization significantly increased antigen-specific TNFα secretion in young animals compared to pS-only immunized young animals while significantly decreasing TNFα secretion in aged animals compared to pS-only immunized aged mice. This resulted in equivalent TNFα secretion among young and aged pADA co-immunized animals. This pattern of cytokine secretion was also evident in the CD4^+^ T compartment. Importantly pADA co-immunization inhibited IL-4 secretion from these cells, resulting in significantly enhanced IFNγ:IL-4 ratios suggestive of a strong T_H_1 response among CD4^+^ T cells. In support of enhanced T_H_2-skewing of responses in aged animals, we observed increased production of IgG1 when aged animals were immunized with pS alone. pADA co-immunization however, supported an increased IgG2c to IgG1 ratio in aged mice, suggesting that pADA supports T_H_1-like immunity in aged mice.

In mouse-adapted SARS-CoV-2 challenge we observed protection from morbidity among all immunized animals. However, when we quantified viral load *via* qPCR, aged animals had significantly higher viral RNA loads compared to young animals when immunized with pS alone. pADA co-delivery supported similarly lowered viral loads among aged and young mice. Aged human ACE2 transgenic mice are not readily available, therefore we aged human ACE2 transgenic mice 68-72 weeks in-house prior to immunization and challenge. In this model we observed increased morbidity as indicated by weight loss among aged animals compared to their young counterparts in both the pS-only and pADA co-immunized cohorts. Interestingly, among aged animals only pADA co-delivery resulted in significantly lowered lung viral loads as measured by TCID50 assay. Survival rates were similar between pS-only immunized aged mice and their pADA co-immunized counterparts. These data suggest that pADA enhances immunogenicity in aged animals and can support lowered lung viral loads post-challenge, however the hACE2 transgenic model wherein CNS infection may lead to enhanced mortality, may not be the most biologically relevant model to characterize such differences (45). A caveat of this study is that it may have been underpowered due to study design limitations and larger studies could investigate this in more detail.

In the context of ChAdOx1 SARS-CoV-2 vaccines in aged mice, a booster immunization enhanced responses, however aged mice still did not achieve equivalent immune responses to their young counterparts (46) as has been reported in human populations receiving SARS-CoV-2 vaccines. Similarly, enhanced immunogenicity and protection against SARS-CoV-2 in aged mice was reported when spike protein antigens were co-delivered with a combination of CpG and alum-based adjuvants (47). This enhancement was associated with increased inflammatory cytokine secretion which we did not observed when aged animals were co-immunized with pADA. Lastly, a recent report demonstrated that increased disease burden in aged mice is due to an impairment in interferon responses and that the aged-disease phenotype can be recapitulated in IFN-deficient mice (48). Here, we observed decreased IFN responses in aged animals which could be enhanced by pADA delivery. Collectively, our data support that pADA can affect challenge outcome and enhance vaccine immunogenicity. In combination with the currently available studies on immune responses to SARS-CoV-2 vaccines in aged mice, these studies suggest the involvement of multiple immune response pathways and indicate that intrinsic age-associated changes in immune function and/or pathogen sensing may be responsible for decreased vaccine immunogenicity and increased COVID-19 morbidity and mortality among the aged.

To define age-associated changes at the molecular level, determine if pADA-mediated enhancement depended upon increased interferon signaling, and identify novel targets which may be exploited to enhance vaccine-induced responses in the aged, we performed scRNAseq analysis on the lymph nodes from young and aged pS-only and pADA co-immunized mice. We observed a clear and dichotomous expression landscape in the aged lymph node. Aged lymph nodes were characterized by inflammatory and hyper-regulatory gene signatures including increases in the T_H_2 pathway, T_reg_ associated genes (*FoxP3*, *CD83*), and exhaustion-associated genes (*PD1*/*PDL1*). Interestingly, when we compared the lymph node gene signatures of pADA co-immunized aged animals with pADA co-immunized young animals, we observed a reversal of this phenotype. pADA co-immunized young and aged lymph nodes had similar inflammatory and regulatory gene signatures. Lastly, we evaluated DEGs in lymph node DCs among aged mice only, and compared pS-only immunized aged DCs to pADA co-immunized aged DCs. Among DCs, pADA co-immunization supported gene expression profile indicative of increased antigen capture and presentation. Ingenuity pathway analysis also confirmed our observation of increased T_H_2-type T cell responses. We observed an upregulation of T_H_2-like gene pathways in pS-only immunized aged mice compared to their young counterparts. When aged animals were co-immunized with pADA we observed a significant inhibition of the T_H_2-like gene pathway compared to young mice.

These studies highlight the age-associated changes in immune response to vaccine antigens and demonstrate that the aged mouse represents a viable model for studying these effects in the context of SARS-CoV-2 vaccination and challenge. scRNAseq analysis suggested an increase in immunoregulation and inflammation in the ageing lymph node may be responsible for the observed decreased vaccine-induced immunity. pADA significantly enhanced vaccine-induced responses and altered gene signatures in aged mice such that we observed similar immune responses and lymph node gene signatures between aged and young pADA co-immunized animals and similar viral loads post-challenge. These studies have implications for the continued design of vaccines targeting not only SARS-CoV-2 but any pathogens which disproportionately affect aged populations.

## Methods

### Resource availability

#### Materials availability

This study generated unique DNA plasmid immunogens and matched peptides as well as ACE2-expressing modified AAV6 vectors and SARS-CoV-2 pseudotyped viruses, all of which are proprietary.

#### Data and code availability

The published article includes all data sets generated or analyzed during this study. Sequencing data was submitted to NCBI GEO database under accession number GSE217200.

#### Plasmids and immunizations

Codon-optimized DNA plasmids encoding full-length SARS-CoV-2 spike glycoproteins were produced commercially and subcloned into the pVax expression vector with an IgE leader sequence to facilitate *in vivo* secretion. Similarly, a synDNA construct encoding murine ADA-1 was generated in the pVax vector and commercially produced (Genscript, Piscataway, NJ) as previously described (12). C57BL/6 male and female mice aged 6-8weeks or 68-72 weeks were immunized twice separated by 4 weeks in the left tibialis anterior muscle with 30-50µL of the formulated vaccines. Vaccines included 10µg of pS alone or, 10ug of pS co-formulated with 10µg of pADA. Control mice remained unvaccinated or were immunized with 20ug of empty plasmid vector (pVax). K18 hACE2 transgenic male and female mice aged 6-8weeks or 68-72 weeks and BALB/c female mice aged 6-8weeks were also immunized as described above. Immediately following vaccine injection, *in vivo* electroporation was performed using the CELLECTRA device (Inovio Pharmaceuticals, Bluebell, PA). All animals were housed in a temperature-controlled, light-cycled, specific-pathogen-free facilities at the Wistar Institute, Drexel University College of Medicine, The university of Pennsylvania, or The public health agency of Canada, and all studies were performed in accordance with approved IACUC protocols.

#### Mouse sacrifice, sample collection and tissue harvest

At the time points shown in the *in vivo* study designs, mice were either bled *via* cheek bleed or sacrificed. At sacrifice, blood, spleens, lymph nodes, and lungs were collected. Blood was collected *via* cheek bleed or cardiac puncture into minicollect serum gel tubes (Grenier-Bio) and centrifuged at 13,000 rpm for 10 min at 4°C to separate serum. Spleens and lungs were processed into single-cell suspensions, washed and resuspended in RPMI medium supplemented with 1% penicillin/streptomycin and 10% FBS. Cell concentrations and viabilities were determined using a Countess Automated Cell Counter (Invitrogen, Life Technologies).

#### SARS-CoV-2 challenge

All SARS-CoV-2 wild-type virus challenges were performed in the animal BSL3 facility at the Public Health agency of Canada under appropriate animal use protocols. For AAV6-mediated ACE2 challenge, BALB/c mice were administered 10^11^ vector genomes of either AAV6.2FF-hACE2 intranasally in 50 ml (25 ml per nare). On day 7 after AAV infection, virus (SARS-CoV-2; hCoV-19/Canada/ON-VIDO-01/2020, GISAID accession #EPI_ISL_425177) was diluted in media, and animals were administered 10^5^ TCID50 intranasally in 50 ml (25 ml per nare). Animals were recovered and then weighed and monitored daily for any clinical signs of disease. On day 4 post infection, animals were euthanized to examine viral replication in the respiratory tract. Euthanasia was performed by anesthesia with inhaled isoflurane followed by cervical dislocation. Human ACE2 transgenic (K18) mice were anesthetized with inhalation isoflurane and were infected with virus (SARS-CoV-2; hCoV-19/Canada/ON-VIDO-01/2020, GISAID accession #EPI_ISL_425177) that was diluted in media. Animals were administered 10^5^ TCID_50_ intranasally in 50 ml (25 ml per nare). Animals were recovered and then weighed and monitored daily for any clinical signs of disease. Mouse adapted SARS-CoV-2 challenge was performed in the animal BSL3 facility at the University of Pennsylvania according to appropriate animal use protocols. C57BL/6 mice were infected with mouse adapted SARS-CoV-2. Animals were administered 1x10^5^ TCID_50_ intranasally in 50 ml (25 ml per nare). Animals were recovered and then weighed and monitored daily for any clinical signs of disease. On day 7 post infection, animals were euthanized to examine viral replication in the respiratory tract. Euthanasia was performed under isoflurane anesthesia.

#### Detection of SARS-CoV-2 in tissues

For measurement of viral titers in the lungs of infected mice, TCID_50_ assays were performed. Following necropsy, tissue samples were frozen at -80°C. Tissue samples were thawed and placed in MEM, supplemented with 1x L-glutamine and 1% FBS and homogenized with 5 mm stainless steel beads in a Bead Ruptor Elite Tissue Homogenizer (Omni). Homogenates were clarified by centrifugation at 1500 x g for 10 minutes and ten-fold serial dilutions of tissue homogenates were made in MEM. Dilutions were added to 90-100% confluent Vero cells in triplicate wells and cytopathic effect was read at 5 dpi. TCID_50_ values per gram of tissue were calculated using the Reed and Muench method.

For detection of viral RNA, tissues collected were stored in RNAlater. RNA was extracted using an RNeasy mini plus kit (Qiagen), according to manufacturer’s instructions. RT-qPCR detection of SARS-CoV-2 was performed on a QuantStudio 5 instrument (Applied Biosystems) using a TaqPath 1-step RT-qPCR Master Mix (Applied Biosystems) and primers specific for the E gene of SARS-CoV-2, as per the diagnostic protocol recommended by the World Health Organization (Forward – ACAGGTACGTTAATAGTTAATAGCGT; Reverse –ATATTGCAGCAGTACGCACACA; Probe–FAM-ACACTAGCCATCCTTACTGCGCTTCGBBQ). Oligonucleotide concentrations were 400nM for the primers and 200nM for the probe. RT-qPCR stages were as follows: uracil-*N*-glycosylase incubation (25°C for 2 minutes), reverse transcription (53°C for 10 minutes), polymerase activation (95°C for 2 minutes), followed by amplification (40 cycles of 95°C for 3 seconds and 60°C for 30 seconds).

#### ELISA assays

ELISA was used to determine RBD specific IgG present in mouse serum. Mouse blood samples were collected *via* cheek bleed or cardiac puncture. Enzyme immunoassay/radioimmunoassay (EIA/RIA) plates (Corning) were coated with 100µL per well of recombinant RBD (Sino Biologicals) that was diluted in PBS to a concentration of 0.5µg/mL. Plates were incubated overnight at 4°C. Plates were blocked using 3% BSA in 1x PBS for 2-4hrs at room temperature. Mouse serum was diluted in PBS with 1% BSA, added in duplicate and incubated overnight at 4°C. Plates were washed three times using PBS with 0.1% tween. HRP conjugated goat anti-mouse IgG (KPL or Columbia Biosciences) secondary antibody was added to plates. Plates were washed three times using PBS with 0.1% tween and were developed using TMB Ultra substrate (Thermo Fisher) according to the manufacturer’s instructions. RBD specific IgG present in the sera was determined by end point titer or by interpolating the optical densities on calibration curves which were generated with known quantities of mouse IgG (Thomas Scientific).

#### ELIspot assays

To quantify interferon gamma (IFNγ) secreting splenocytes/lymphocytes, mouse IFN-γ ELISpot plates (Mabtech, Cincinnati, OH) were used according to the manufacturer’s recommended protocol. Briefly, plates were washed four times with sterile PBS, followed by a two-hour incubation with R10 media for blocking. The plate was seeded with 200,000 cells in duplicate suspended in 100µL R10. Cells were stimulated with 5µg/ml of matched overlapping SARS-CoV-2 spike peptide pools representing the entire spike protein, or DMSO and phorbol myristate acetate/ionomycin (PMA/Iono) as negative and positive controls respectively. After incubation at 37°C in 5% CO_2_ for 18 hours, plates were developed following the manufacturer’s protocol. Plates were scanned and counted using the CTL ImmunoSpot S6 Universal Analyzer (Cellular Technology Limited, Shaker Heights, OH).

#### Surface plasmon resonance assays

SPR experiments were performed on a Biacore S200 biosensor (Global Cytiva Lifesciences) at 25°C using PBS-P (10 mM Phosphate, 150 mM NaCl, pH 7.4, 0.05% P-20) as the running buffer. A CM5 sensor chip (Cytiva, Marlborough, MA) was docked and derivatized by amine coupling with RBD using freshly prepared 100 mM NHS (N-hydroxysuccinamide) and 400 mM EDC (1-ethyl-3-(3-(dimethylamino) propyl) carbodiimide) reagents (Cytiva) mixed 1:1. Flow cell 1 was activated and blocked and remained as the control of binding to RBD (Purified Wuhan recombinant Spike RBD, Sino Biological, BDA, Beijing, RBD-His 40592-VNAH, FC2), S1 (S1-His 40150-V08B1), and S2 (S2-mfc 40590-V05B). S2-His 40590-V05B expressed in insect cells was used in serum antibody subtype characterization. RBD and S1 activities were validated using ACE2 10108-H08H. The anti-spike titration standard was either a RBD (40592-MM57), S1 (40591_MM43) or S2 (40590-D001) specific mouse monoclonal antibody (Mab); and VHH-72-huFc a generous gift from Integral Molecular (Philadelphia, PA). Serum from post-immunization bleeds were diluted in sample buffer with 20 mg/mL CM-Dextran saline, or NSB reducer (Cytiva) to minimize non-specific binding, as previously reported (Cusimano et al, 2022). Sample matching naïve sera mixed with CM-Dextran in equal proportion, were used as negative controls, while Mab standards between 1.2 to 250 nM were also spiked in naïve serum mixed CM-Dextran. Raw data gathering was blinded to sample identity. A surface density of 50 RU/KDa RBD was experimentally found to offer partial mass transport limited (MTL) binding of serum-spiked antibody standards injected over all spike domains under MTL. Concentrations of active, specific antibodies binding RBD, S1, and S2 were calculated as reported by Cusimano et al, (2022). The kinetics of pAb binding to RBD, S1, and S2, were obtained by injecting dilute sera over all surfaces in duplicate at flow 50 µL/min All binding profiles (sensorgrams) were double referenced to minimize the impact of instrument and solvent noise. Two types of calculations were carried out to fully characterize individual sera: pAb epitope specific concentration (dR/dt=kt*C) and binding kinetics (dR/dt= ka*C*(Rmax-R)- kd*R), where Rmax and R were measured in RU (response units), C was the antibody concentration, kt was the mass transport constant, ka and kd were the kinetic association and dissociation constants. Specific pAb concentrations were calculated using Phenom v0.7.202101-alpha (Meritoki). Standard Mab binding profiles were globally fit to a 1:1 binding model to calculate the kinetic constants ka and kd and maximum binding capacity for each domain surface (or Rmax) using Scrubber 2.c (BioLogic Software, AU). In the same way, the profiles of pAb binding to RBD were analyzed by fixing the Rmax to the previously determined binding capacity for the RBD surface. The average kinetic parameters generated from two independent datasets (1/15 and 1/20 dilutions) were identical and were used to calculate the equilibrium association constants KA (ka/kd) M-1.

#### T cell flow cytometry

Single cell suspensions of splenocytes from vaccinated mice were cultured for 6 hours in the presence of five SARS-CoV-2 spike peptide pools [5 ug/mL final concentration] or in a 1x working dilution of Cell Activation Cocktail (BioLegend) (Cat: 423301) in 96-well U bottom plates at a concentration of 10^6^ cells per well. As a negative control, cells were treated with an equal percentage amount of DMSO. All samples were also treated with a protein transport inhibitor (eBioscience) (Cat: 00-4980-93) for the duration of the 6 hour stimulation. Following stimulation, cells were stained with the live/dead Fixable Aqua Dead Cell Kit (Invitrogen) (dilution 1/100; Cat: L34957) to gate on live cells and anti-mouse CD16/32 antibody (BioLegend) (Clone: 93; Cat: 101319). Cells were then stained at 4°C for 30 minutes with the following fluorochrome-conjugated anti-mouse antibodies: CD4 (Clone: GK1.5; Cat: 100438), CD8 (Clone: 53-6.7; Cat: 100714), IL-4 (Clone: 11B11; Cat: 504133), IFN-γ (Clone: XMG1.2; Cat: 505810), TNF-α (Clone: MP6-XT22, Cat: 506346), CD3 (Clone: 145.2C11; Cat: 100310), PD-1 (Clone: RMP1-30; Cat: 109110), CXCR5 (Clone: 2G8; Cat: 560615), CD154 (Clone: MR1 Cat: 106510) FoxP3 (Clone : MF-14 Cat: 126422). All cytometric analyses were performed using an LSR II flow cytometer (BD), and data were analyzed using FlowJo software (Treestar).

#### Neutralization assays

Pseudoviruses neutralization assays were performed as previously described (13). For live virus neutralization, isolates were received from BEI resources. Live virus neutralization assays were performed in the Wistar Institutes BSL3 facility according to approved SOPs. Live neutralization assays were performed as previously described (49). Briefly, virus was combined with diluted sera samples and incubated 1 h at 37 °CC and then adsorbed onto ATCC Vero 76 cell monolayers in 96-well tissue culture plates. After 1 h at 37 °CC in a 5% CO2 incubator, virus-serum mixtures were added to 96-well plates containing Vero cells in triplicate and incubated for 3- 5 days with daily observations for cytopathic effect (CPE).

### Quantification and statistical analysis

#### Statistical analysis

All statistics were analyzed using GraphPad Prism 9. Error bars represent means ± SEM, mean ± SD, or geometric means ± geometric SD where noted. Normality was determined using the Shapiro Wilk normality test. Outliers were determined and removed using the robust regression and outlier removal algorithm. For data deemed normal, ordinary one-way ANOVA was performed to determine statistical significance between groups of three or more (Tukey’s multiple comparison test). For data deemed non-normal, a nonparametric Kruskal Wallis test was performed to determine statistical differences between groups of three or more. Statistical differences between groups of two were determined *via* unpaired t test for data deemed normal, or Mann Whitney U test for data deemed non-normal as indicated. In all data, *p < 0.05, **p < 0.01, ***p < 0.001, and ****p < 0.0001.

#### Bioinformatics analysis of scRNA-Seq

Preprocessing of scRNA-seq data was done using CellRanger Suite (v3.1.0, https://support.10xgenomics.com ) with refdata-cellranger-mm10-3.0.0 transcriptome as a reference to map reads on mouse genome (mm10) using STAR (50). Seurat R package (51) was used for further processing and analysis. Cells with less than 200 genes with reads and over 10% mitochondrial content were discarded owing to poor quality. The remaining 49,848 cells distributed across the 6 samples were subjected to normalization and sample integration to account for batch effect. Seurat was also used for cell clustering, identification of markers, and visualization. An R package SingleR (51, 52) was used to predict cell types using ImmGen data set as a reference set (53) for gene signatures specific to cell types. Further resolution of cell types was then carried out using known gene markers associated with different cell sub-types. Differentially expressed genes between aged and young samples across treatment groups as well as between treatment groups in different cell types was carried out using non-parametric Wilcoxon rank sum test and genes that passed FDR < 5% were used as inputs for pathway analysis using QIAGEN’s Ingenuity Pathway Analysis (IPA^®^, QIAGEN Redwood City, www.qiagen.com/ingenuity ) unless stated otherwise. Data was submitted to NCBI GEO database under accession number GSE217200.

## Data availability statement

The data presented in the study are deposited in the Genebank (https://www.ncbi.nlm.nih.gov/genbank/) repository, accession number GSE217200.

## Ethics statement

The animal study was reviewed and approved by The Wistar Institute IACUC committee.

## Author contributions

EG, NT, BW, GC, EH, MK, and DW designed experiments. EG, NT, GC, and MB performed animal immunizations, blood collection and assays. EG, NT, JC, EP, BG, AA, DF, CEH, and GC, performed immunological assays. BW, BG, and DK performed containment challenge and viral reproduction assays. GAC performed SPR experiments. SW produced AAV vectored ACE2, TK and AK performed bioinformatics analysis. The manuscript was written by EG, NT, and GC, with intellectual oversight from IC, AP, MK, EH, and DW. The manuscript was submitted on behalf of all authors by EG and DW. All authors contributed to the article and approved the submitted version.

## Supplementary material

Supplementary Figure 1 Co-immunization with pADA enhances SARS-CoV-2 cellular responses in aged mice. Frequencies of cytokine positive CD8+ **(A-B)** and CD4+ **(C–F)** in young mice only. Frequencies of cytokine positive CD8+ **(G–H)** and CD4+ **(I–L)** in aged mice only. ​

Supplementary Figure 2 Co-immunization with pADA enhances protection from SARS-CoV-2 challenge in young mice. Female BALB/c mice 6-8 weeks old were immunized once or twice separated by 28 days with 10µg of pS or 10µg of pS with 10µg of pADA (+pADA). Serum was collected at day 14 post first immunization and day 14 post-2^nd^ immunization. **(A)**. Serum RBD specific IgG was quantified *via* ELISA at day 14 post-1^st^ immunization **(B)**, and day 14 post-2^nd^ immunization **(C)** post immunization. Young and aged C57BL/6 animals were immunized and twice and bled as in A **(D)** and SARS-CoV-2 pseudotyped virus (USA-WA1/2020) neutralization was quantified at day 14 post-1^st^ immunization **(E)** and Day 14 post-2^nd^ immunization **(F).**Each point represents an individual animal. Bars represent the mean and error bars represent the SD. *p<0.05, **p<0.01, ***p<0.001, and ****p<0.0001 as measured by Kruskal-Wallis ANOVA **(B, C)** or Mann-Whitney U test **(E, F)**. Data are representative of 4 experiments **(B)**, 5 experiments **(C)** or 1 experiments **(E, F)** with n= 5-8 mice per group.

Supplementary Figure 3 scRNAseq identifies distinct cellular subsets and characterizes age-associated genomic changes. Mice were immunized twice with pS alone or co-immunized with pADA as in and popliteal and inguinal lymph nodes from 10 mice per immunization condition were pooled and subjected to single-cell RNA sequencing (scRNAseq) after specific filtering criteria **(A).** Representative **(B)** Seurat clustering and **(C)** singleR cell subset analysis on draining lymph nodes. **(D)** Representative re-clustering of CD4+ T cells to resolve FOXP3+ (right), follicular helper T (middle) cells for differential gene expression analysis. Select differentially expressed genes in aged animals compared to young animals among CD8^+^ **(E)**, CD4^+^ **(F)** T cells, and B cells **(G).**

Supplementary Figure 4 scRNAseq identifies disti(nct cellular subsets and characterizes age-associated genomic changes. Mice were immunized twice with pS alone or co-immunized with pADA as in and popliteal and inguinal lymph nodes from 10 mice per immunization condition were pooled and subjected to single-cell RNA sequencing (scRNAseq). Representative **(A)** Seurat clustering and **(B)** singleR cell subset analysis on draining lymph nodes. **(C)** Representative re-clustering of CD4+ T cells (right) to resolve FOXP3+ (left), follicular helper T (middle) cells for differential gene expression analysis. Select differentially expressed genes in aged animals compared to young animals among CD8^+^ **(D)**, CD4^+^ **(E)** T cells, and B cells **(F)**.
